# Diet-Induced Obesity Elicits Macrophage Infiltration and Reduction in Spine Density in the Hypothalami of Male but Not Female Mice

**DOI:** 10.3389/fimmu.2018.01992

**Published:** 2018-09-11

**Authors:** Nancy M. Lainez, Carrie R. Jonak, Meera G. Nair, Iryna M. Ethell, Emma H. Wilson, Monica J. Carson, Djurdjica Coss

**Affiliations:** Division of Biomedical Sciences, School of Medicine, University of California, Riverside, Riverside, CA, United States

**Keywords:** sex-specific, cytokine, GnRH, neuroinflammation, obesity, hypothalamus

## Abstract

Increasing prevalence in obesity has become a significant public concern. C57BL/6J mice are prone to diet-induced obesity (DIO) when fed high-fat diet (HFD), and develop chronic inflammation and metabolic syndrome, making them a good model to analyze mechanisms whereby obesity elicits pathologies. DIO mice demonstrated profound sex differences in response to HFD with respect to inflammation and hypothalamic function. First, we determined that males are prone to DIO, while females are resistant. Ovariectomized females, on the other hand, are susceptible to DIO, implying protection by ovarian hormones. Males, but not females, exhibit changes in hypothalamic neuropeptide expression. Surprisingly, ovariectomized females remain resistant to neuroendocrine changes, showing that ovarian hormones are not necessary for protection. Second, obese mice exhibit sex differences in DIO-induced inflammation. Microglial activation and peripheral macrophage infiltration is seen in the hypothalami of males, while females are protected from the increase in inflammatory cytokines and do not exhibit microglia morphology changes nor monocyte-derived macrophage infiltration, regardless of the presence of ovarian hormones. Strikingly, the anti-inflammatory cytokine IL-10 is increased in the hypothalami of females but not males. Third, this study posits a potential mechanism of obesity-induced impairment of hypothalamic function whereby obese males exhibit reduced levels of synaptic proteins in the hypothalamus and fewer spines in GnRH neurons, located in the areas exhibiting macrophage infiltration. Our studies suggest that inflammation-induced synaptic remodeling is potentially responsible for hypothalamic impairment that may contribute to diminished levels of gonadotropin hormones, testosterone, and sperm numbers, which we observe and corresponds to the observations in obese humans. Taken together, our data implicate neuro-immune mechanisms underlying sex-specific differences in obesity-induced impairment of the hypothalamic function with potential consequences for reproduction and fertility.

## Introduction

Over half of the US population is classified as overweight and a full third is classified as obese (1). The number of obese people has increased steadily over the last 30 years (2). This increase in obesity has coincided with an increase in co-morbidities, such as type 2 diabetes, cardiovascular disease, stroke, and hypothalamic disorders, including reproductive disorders that cause infertility (3–6). Deleterious effects of obesity on fertility include irregularities in menstrual cycles, abnormalities in the oocyte development, anovulation, and increased risk of miscarriages in women (3); and inferior sperm quality, reduced sperm quantity, and lower testosterone levels in men (7). Currently, 18% of couples require assisted reproductive technologies to become pregnant (8), a portion of which may stem from widespread obesity (9, 10). Although several hypotheses have been put forth (11–15), mechanisms whereby obesity negatively affects reproductive function are unknown.

The hypothalamus in the basal forebrain controls feeding and satiety, thermoregulation, thirst, circadian rhythms, metabolism, and mammalian reproduction. In the control of reproduction, the hypothalamic decapeptide gonadotropin-releasing hormone (GnRH) is the final brain output that regulates expression and secretion of gonadotropins, luteinizing hormone (LH) and follicle stimulating hormone (FSH) from the anterior pituitary gonadotropes (16), which in turn stimulate steroidogenesis and gametogenesis in the gonads (17, 18). In the rodent hypothalamus, the majority of GnRH cell bodies are scattered in the preoptic area surrounding organum vasculosum laminae terminalis (OVLT), while secretion occurs at the terminals in the median eminence (ME). Both OVLT and ME are circumventricular areas with a leaky blood-brain barrier, whereby hypothalamic neurons perceive changes in the circulation and neuropeptides reach the portal circulation (19). GnRH secretion is synchronized by upstream regulatory neuronal populations (20). These regulatory neurons integrate sex steroid feedback from the gonads (21), and external or environmental factors, such as stress (22, 23), and exposure to endocrine disruptors (24, 25). Metabolic stimuli and energy status, such as anorexia nervosa, excessive exercise, malnutrition, and obesity are also integrated with reproductive function at the level of the hypothalamus (11–13, 15, 26, 27). However, since metabolic signals do not influence GnRH neurons directly (28–30), we investigated other potential mechanisms.

Obesity is characterized by chronic inflammation, in addition to changes in metabolic markers (31). Increased adiposity elicits an increase in inflammatory cytokines in the circulation (32, 33), such as: tumor necrosis factor (TNFα), and interleukins, IL-1β, IL-6 (34), primarily due to macrophage infiltration to adipose tissue and their subsequent activation. Inflammatory cytokines have been demonstrated to negatively affect reproductive function (35). Influence of acute inflammation on reproduction was an area of intense investigation and these studies determined that LPS or cytokine administration in the brain ventricle reduces gonadotropin levels, diminishes GnRH neuropeptide release and represses GnRH and LH gene expression (36–38). As opposed to the acute, high level of inflammatory cytokines used in previous studies, obesity elicits low grade, chronic inflammation and we investigated its effects on reproduction via GnRH neurons.

To analyze the effects of obesity-induced inflammation on reproductive function we used diet-induced obese (DIO) mice. Significant strain differences were observed in response to high-fat diet (HFD) and A/J, FVB/NJ and BALB/cJ strains are resistant to DIO, while DBA/2J and C57BL/6J gain weight (39–41). The C57BL/6J mouse is a particularly faithful model of the human metabolic syndrome because it develops obesity, hyperinsulinemia, hyperglycemia, and hypertension, when allowed *ad libitum* access to a HFD (42, 43). Herein, we demonstrate profound sex differences in response to HFD. Specifically, C57BL/6J male mice exhibit neuroinflammation, with a resultant decrease in the number of synaptic spines on GnRH neurons and reduction in GnRH mRNA levels. On the other hand, female mice are resistant to neuroendocrine and inflammatory changes, and this protection is independent of ovarian hormones. Together, our data implicate sex-specific effects in obesity-induced neuroinflammation with functional consequences on GnRH neurons.

## Materials and methods

### Animals

C57BL/6J mice were obtained from Jackson Laboratory at 3 weeks of age. After a week acclimatization, they were randomly assigned to the high-fat diet fed group (HFD, D12492, 60% kcal from fat; Research Diet, New Brunswick, NJ) and control group (Ctr, D12450J, 10% kcal; Research Diet, New Brunswick, NJ) for an indicated number of weeks. Animals were maintained under a 12-h light, 12-h dark cycle and received food and water *ad libitum*. All experiments were performed with approval from the University of California Animal Care and Use Committee and in accordance with the National Institutes of Health Animal Care and Use Guidelines using 16-week-old animals (3 weeks before weaning, 1 week normal chow, 12 weeks high fat or control diet) unless indicated otherwise. During the week between weaning and experimental diet, while fed normal chow, all animals were handled daily by experienced personnel to assure habituation and minimize stress (44).

For fluorescently labeled microglia, CX3CR1-GFP mice were obtained from Jackson labs (strain 005582) and randomly placed on the respective diets at 4 weeks of age to analyze change in microglia morphology. Doubly fluorescent GFP and RFP transgenic mice obtained after crossing CX3CR1-GFP (strain 005582) and CCR2-RFP (strain 017586), and heterozygous mice for both alleles were used to distinguish monocyte recruitment to the hypothalamus from resident microglia. GnRH-GFP mice were kindly provided by Dr. Suzanne Moenter (45). Males and females were analyzed separately to determine sex differences. At least 10 animals per sex per genotype were analyzed, unless indicated otherwise for a specific analysis in the materials and methods section. Statistical differences (*p* < 0.05) between control (Ctr) and high fat diet (HFD) fed mice were determined by Student's *T*-test or 2-way ANOVA where appropriate, and Tukey's test for multiple comparison.

#### Estrous cyclicity

Starting at 12 weeks of the Ctr or HFD, female mice were assessed for estrous cycle stage with daily vaginal smears for 6 weeks. Vaginal lavage was performed daily (between 9-10 am) by flushing the vagina with distilled H_2_O. Collected smears were mounted on glass slides and examined microscopically for cell types. Both groups of animals were handled daily, at the same time of day, to account for any potential stress that handling may cause. Estrous cycle stages determined during the first week of vaginal smears were not included in the analysis to allow for acclimatization. Female mice on control diet exhibited normal 4-5 day-long estrous cycles, indicating habituation to handling. Estrous cycle length was calculated as the length of time between two successive occurrences of estrus. For subsequent studies, females were estrous cycle staged and tissue samples collected in diestrus between 9 and 11 a.m.

#### Sperm count

After 12 weeks on the respective diet, males were sacrificed between 9 and 11 a.m. The epididymides were dissected, macerated, and then incubated in 1 ml DMEM at room temperature for 30 min with shaking. Sperm was cleared with a 70 μm cell strainer, diluted with sterile water and counted with a hemocytometer.

### Cytokine and hormone analyses

For serum collection, mice were sacrificed between 9 and 11 a.m. by isoflurane inhalation and blood was obtained from the inferior *vena cava*. The blood was left to coagulate for 15 min at room temperature, and then centrifuged at 2000 RCF for 15 min for serum separation. Cytokine levels in serum and hypothalamic protein lysates were measured using Luminex MagPix instrument and mouse ProcartaPlex 7 plex (Affymetrix eBioscience, San Diego, CA). Hormone assays were performed by the University of Virginia, Ligand Core. The University of Virginia Center for Research in Reproduction Ligand Assay and Analysis Core is a fee-for-service core facility and is supported in part by the Eunice Kennedy Shriver NICHD/NIH U54-HD28934 Grant. LH was measured by the ultra-sensitive mouse LH ELISA, an in-house method. The capture monoclonal antibody (anti-bovine LH beta subunit, 518B7) is provided by Janet Roser, University of California. The detection polyclonal antibody (rabbit LH antiserum, AFP240580Rb) is provided by the National Hormone and Peptide Program (NHPP). HRP-conjugated polyclonal antibody (goat anti-rabbit) is purchased from DakoCytomation (Glostrup, Denmark; D048701-2). Mouse LH reference prep (AFP5306A; NHPP) is used as the assay standard. Intra-assay coefficient of variation is 2.2% and inter-assay coefficient of variation is 7.3% at the low end of the curve. Functional sensitivity is 0.016 ng/ml. FSH was assayed by RIA using reagents provided by Dr. A.F. Parlow and the National Hormone and Peptide Program, as previously described (46). Mouse FSH reference prep AFP5308D was used for assay standards. Steroid hormone levels were analyzed using validated commercially available assays, information for which can be found on the core's website: http://www.medicine.virginia.edu/research/institutes-and-programs/crr/lab-facilities/assay-methods-page and reported in Haisenleder et al. (47). Limits of detection were 2.4 ng/ml for FSH, 3 pg/ml for estradiol, and 10 ng/dL for testosterone. Intra- and inter-assay coefficients of variation were 6.9%/7.5%, 6.0%/11.4%, and 4.4%/6.4% for the FSH, estrogen (E2) and testosterone (T), respectively. Ten animals per group were used for each hormone analysis. Statistical differences in hormone levels between Ctr and HFD groups were determined by Student's *T*-test, and Tukey-Kramer HSD for multiple comparisons using JMP software (SAS Institute; Cary, North Carolina).

### Flow cytometry

Antibodies used for flow cytometry, immunohistochemisty and western blotting are listed in Table 1. Microglial activation and immune cell influx into the hypothalamus and prefrontal cortex were characterized as previously described (48). Tissues from the hypothalamus and cortex from each mouse was processed separately as part of a 5-mouse cohort per group, with each experiment repeated 3 times. In brief, mice were perfused with ice cold PBS, brains rapidly removed and hypothalami and prefrontal cortex cell suspensions were generated by mechanical dissociation and applied to a discontinuous 1.03/1.088 percoll gradient. Cells were collected from the interface, blocked with anti-CD16/CD32 (1:300, 553141, BD Biosciences, San Jose, CA), and incubated with anti-CD45 APC-eFluor® 780 (1:300, 47-0451, eBioscience, San Diego, CA) and anti-CD11b PerCP-Cyanin5.5 (1:300, 45-0112, eBioscience, San Diego, CA) in PBS, 5% EDTA, 0.4% BSA. Sytox™ Green dead stain (30 nM, S-34860, ThermoFisher, Chino, CA) was used to exclude dead cells and flow analysis performed using BD LSR II Flow Cytometer. Results were analyzed using FlowJo software (Tree Star, Inc.) and statistical differences were determined by Student's T-test and Tukey's posthoc test.

**Table 1:** Antibodies.

**Antibody**	**Species**	**Dilution**	**Provider, cat # and RRID**
GFP	chicken	1:5,000	Abcam, ab13970; **AB_300798**
Iba-1	rabbit	1:300	Wako, 019-19741; **AB_839504**
PSD-95	rabbit	1:2,000	Cell Signaling, 3409; **AB_1264242**
Synaptophysin (SYPH)	rabbit	1:1,000	Cell Signaling, 4329; **AB_1904154**
MAP2	chicken	1:5,000	Abcam, ab5392; **AB_2138153**
GnRH	rabbit	1:1,000	ThermoFisher, PA1-121; **AB_325077**
β-tubulin	rabbit	1:1,000	Santa Cruz Biotechnology, sc-9104; **AB_2241191**
CD45 APC-eFluor 780	rat	1:300	eBioscience, 47-0451 (clone 30-F11), **AB_1548781**
CD11b PerCP-Cy5.5	rat	1:300	eBioscience, 45-0112 (clone M1/70), **AB_953558**

*Bold indicates: RRID, RESEARCH RESOURCE IDENTIFIERS (RRIDs) number*.

### Histological analyses and immunohistochemistry

Following Ctr or HFD mice were anesthetized, perfused with 20 ml PBS and 20 ml 4% paraformaldehyde; and tissues collected. Brains were fixed in 4% paraformaldehyde, embedded in paraffin, and cut to 20 μm. Slides were deparaffinized in xylene and rehydrated. Antigen unmasking was performed by heating for 10 min in a Tris-EDTA-0.3% Triton X and endogenous peroxidase was quenched by incubating for 10 min in 0.3% hydrogen peroxide. Slides were then blocked with 20% goat serum and incubated with primary antiserum against GnRH (1:1000 PA1-121, Thermo Sci.) or Iba-1 for microglia (1:300 cat # 019-19741, Wako) overnight at 4°C. After PBS washes, slides were incubated with biotinylated goat anti-rabbit IgG (1:300, BA-1000, Vector Laboratories, Burlingame, CA) for 30 min. The Vectastain ABC elite kit (Vector Laboratories) was used per manufacturer's instructions, after which the DAB peroxidase kit was used for colorimetric staining. Slides were dehydrated in ethanol and xylene, and cover-slipped with Vectamount (Vector Laboratories).

To visualize GFP-labeled microglia from CX3CR1-GFP mice, and activated macrophages labeled red and microglia labeled green from CCR2-RFP x CX3CR1-GFP mice, after 12 weeks of Ctr or HFD mice were anesthetized, perfused with 20 ml PBS followed by 20 ml of 4% paraformaldehyde; brains were post-fixed in 4% paraformaldehyde, frozen in OCT, and cut to 20 μm sections using Leica cryostat. Endogenous fluorescence was visualized with Leica microscope.

Hypothalami from GnRH-GFP mice were sectioned to 100 μm sections and sections containing organum vasculosum laminae terminalis (OVLT) where GnRH neurons are located, stained for GFP to visualize GnRH neurons. Slides were blocked with 20% goat serum and incubated with primary antibodies against GFP (1:5000 raised in chicken), at 4°C for 48 h. After PBS washes, slides were incubated with FITC/Alexa 488 goat anti-chicken IgG (1:300, Molecular Probes, Eugene, OR) for 30 min. Secondary antibody-only controls were performed to determine antibody specificity. Images were obtained using confocal Leica SP2 microscope. To determine spine density, we followed our established protocol as published before (49–53), with modifications that correspond to previously described procedures for analysis of spines in GnRH neurons (54, 55). Spines, that were identified as protrusions from the soma or axon greater than 1 μm, were counted in the individual neurons where at least 75 um of the axon proximal to soma can be observed using z-stack. The full length of every GFP-labeled GnRH neuron, including soma and the visible length of the main process, was imaged and analyzed using confocal Leica SP2 microscope. Images were encoded for blind analysis. Spine numbers were quantified by scrolling through the series of captured images in the z-stack using LAS X software and counted for each GFP-labeled GnRH soma and along the 75 μm length of axon, at 15-μm intervals. We counted at least 3 individual neurons from 4 different sets of mice marked by hand and using Neurolucida program (MBF Bioscience, Vermont).

### Western blot

Whole cell lysates were obtained from the dissected hypothalami from Ctr and HFD fed mice and after protein determination, the same amount of protein was run on SDS-PAGE, transferred on nitrocellulose membrane and probed for: Postsynaptic density protein 95 (PSD-95; 1:1000, Cat #3409, Cell Signaling), Synaptophysin (SYPH; 1:1000, cat. #4329, Cell Signaling), neuronal marker, Microtubule-associated protein 2 (MAP2; 1:5000, cat #ab5392, Abcam) or β-tubulin (1:1000, cat #sc-9104, Santa Cruz Biotechnology). Bands were quantified using ChemiDoc imaging system (Bio-Rad, Hercules, CA).

### qPCR analyses

Hypothalami were dissected, total RNA extracted and reverse transcribed using Superscript III (Invitrogen, CA). qPCR was performed using an iQ SYBR Green supermix and an IQ5 real-time PCR machine (Bio-Rad Laboratories, Hercules, CA) with primers listed in Table 2 under the following conditions: 95°C for 15 min, followed by 40 cycles at 95°C for 20 s, 56°C for 30 s, and 72°C for 30 s. The amount of the gene of interest was calculated by comparing the threshold cycle obtained for each sample with the standard curve generated in the same run. Replicates were averaged and divided by the mean value of the beta-2-microglobulin (B2M) housekeeping gene in the same sample using ^ΔΔ^Ct method. Preliminary studies analyzing GAPDH, Ywaz, TBP and B2M housekeeping genes determined that B2M doesn't change with diet and was subsequently used for normalization. After each run, a melting curve analysis was performed to confirm that a single amplicon was generated in each reaction. Statistical differences in expression between genotypes were determined by Student's *T*-test, and Tukey's HSD for multiple comparisons using JMP software (SAS Institute; Cary, North Carolina).

**Table 2:** Primers.

**Primers**	**Forward**	**Reverse**
*Gnrh* (GnRH)	CTACTGCTGACTGTGTGTTTG	CATCTTCTTCTGCCTGGCTTC
*Avp* (AVP)	ACACTACGCTCTCCGCTTGT	CGAAGCAGCGTCCTTTGC
*Pomc* (POMC)	CAGTGCCAGGACCTCACC	CAGCGAGAGGTCGAGTTTG
*Il6* (IL-6)	TTCTCTGGGAAATCGTGGAAAT	TCCAGTTTGGTAGCATCCATCA
*Tnfa* (TNFα)	ATGTCTCAGCCTCTTCTCATTCC	GCTTGTCACTCGAATTTTGAGAA
*Lif* (LIF)	ATGTGCGCCTAACATGACAG	TATGCGACCATCCGATACAG
*Il10* (IL-10)	GCTGGACAACATACTGCTAACC	ATTTCCGATAAGGCTTGGCAA
*B2m* (beta-2-microglobulin)	TGACCGGCCTGTATGCTATCCA	CAGTGTGAGCCAGGATATAGAAAGAC
*Gapdh*	TGCACCACCAACTGCTTAG	GGATGCAGGGATGATGTTC

## Results

### Male mice on high-fat diet exhibit lower levels of reproductive hormones

**Figure 1:**
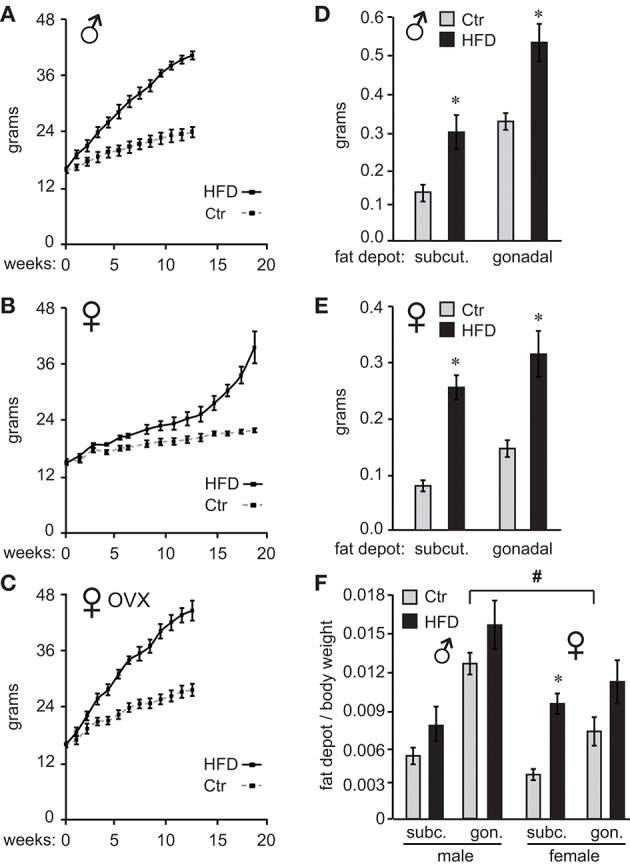
Ovarian hormones provide resistance to diet-induced obesity in female C57Black/6J mice. Ten C57BL/6J mice per group were placed on control (Ctr,10 kcal%fat, Research Diet) or high fat diet (HFD, 60 kcal%fat Research Diet) with the same sucrose levels, at 4 weeks of age. Their weights were recorded twice a week **(A)**, males; **(B)**, females. **(C)**, ♀ OVX, female mice were ovariectomized at weaning and one week later, at 4 weeks of age placed on diets. **(D)**, following exposure to their respective diets, mice were sacrificed and subcutaneous (subcut.) fat depots and gonadal fat depots removed from one side of each animal and weights recorded **(D)**, males; **(E)**, females. **(F)**, Subcutaneous (subc.) and gonadal (gon.) fat depot weight from **(D,E)** was normalized to the whole body weight for each animal. *Indicates significant difference *p* < 0.05 between Ctr (gray bars) and HFD (black bars), while # sign indicates difference between sexes; determined by ANOVA followed by Tukey's HSD test.

C57BL/6J male and female mice were placed on high-fat diet (HFD) or control diet (Ctr) 1 week after weaning and their weights measured twice a week. As demonstrated previously (26), male mice gained weight on HFD, while female mice were resistant to diet-induced obesity (DIO) and required longer exposure to HFD to exhibit the same weight difference from mice on control diet (Figure 1A, male; Figure 1B, female). Preliminary studies indicated that females exposed to the HFD for the same length of time as males do not exhibit adverse effects of obesity illustrated bellow, while much longer exposure would increase the risk of confounding our results with negative effects of aging; thus, we opted to keep the females on the HFD until they reach similar weight gain as males. To determine if ovarian estrogens, or other ovarian hormones, play a role in this sex difference, we ovariectomized (OVX) the females at 4 weeks of age and placed them on the HFD and Ctr. OVX females became susceptible to DIO (Figure 1C). These results indicate that ovarian hormones are protective of DIO.

After 12 weeks on their respective diets for males and 19 weeks for unmodified females, when mice on HFD reached 175% of the weight observed in mice on Ctr diet, we analyzed the adipose tissue deposition by measuring fat depots. We dissected inguinal fat pad from subcutaneous depots, and gonadal fat pad from visceral fat depots, from one side of each animal. Fat pad weights were then compared between Ctr and HFD in both males and females. Weight comparison of fat pads from Ctr and HFD-fed mice indicates that both males (Figure 1D) and females (Figure 1E) deposit fat in both subcutaneous (inguinal) and gonadal fat depots, since fat pads from either depot were heavier in HFD than controls animals. When we compared males to females, Ctr males had significantly heavier subcutaneous fat pads and gonadal fat pads than Ctr females (compare Figures 1D,E). HFD fed males had significantly heavier gonadal fat pads than HFD females. Given the individual weight difference and profound sex differences in size, we also compared fat depots after normalizing fat pad weight to total body weight. There was a significant difference in normalized weight between gonadal fat pads in Ctr males compared to gonadal fat pads in Ctr females, indicating that male mice fed low-fat control diet exhibit higher visceral adiposity than females (Figure 1F, indicated with a pound sign). We did no detect sex differences between other fat depots. Comparison of fat pads between control and HFD revealed preferential fat accumulation in select depots. Significantly, there was a difference in subcutaneous fat pad from HFD female mice compared to subcutaneous fat pad from Ctr females, even after normalizing to the body weight (Figure 1F, asterisk), implying that females preferentially increase adiposity in the subcutaneous depot. Therefore, examination of fat pads may demonstrate that male mice have higher visceral adiposity in control conditions, which corresponds to observations in humans. On the other hand, female obesity may lead to preferential increase in subcutaneous fat, which exhibits less adverse health outcomes (56).

**Figure 2:**
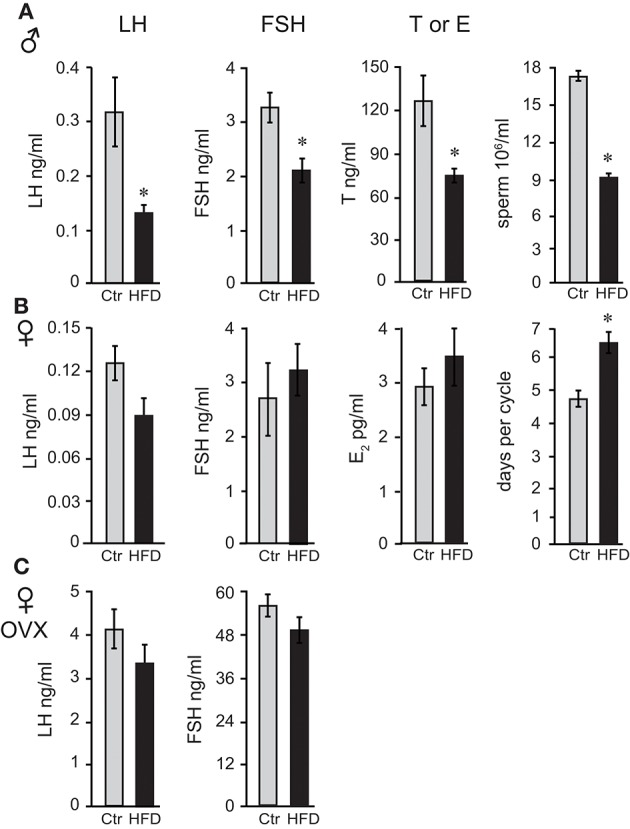
Reproductive hormones are lower in obese male mice, while both unmodified and ovariectomized females are protected from diet-induced changes. Male mice **(A)** on the HFD have reduced levels of luteinizing hormone (LH), follicle-stimulating hormone (FSH) and intra-testicular testosterone (T), and fewer sperm. Female mice **(B)** lack changes in LH, FSH, and estrogen (E_2_), but exhibit longer estrous cycles. Ovariectomized (OVX) females **(C)** are protected from hormonal changes similarly to unmodified females, indicating that the ovarian hormones are dispensable for protection. Differences (*p < 0.05) between control (Ctr, gray bars) and high fat diet (HFD, black bars) were determined by Student's *T*-test followed by Tukey's HSD test.

Following 12 weeks on diets for males and OVX females, which gain weight at the same rate as males, and 19 weeks for unmodified females, we measured gonadotropin hormone and sex steroid hormone levels. Male mice on HFD exhibited 57% lower luteinizing hormone (LH), 35% lower follicle-stimulating hormone (FSH), and 40% lower intra-testicular testosterone levels (Figure 2A). Consequently, HFD males had 50% fewer sperm in their epididymides (Figure 2A), 22% lower seminal vesicle weight, which is dependent on the level of testosterone, while testis weight was unaffected (data not shown). Female mice exhibited longer estrous cycles (4.6 days, controls and 6.4 days, HFD), but LH, FSH or estradiol level (E_2_) in diestrus were not significantly changed (Figure 2B). Thus, female mice, although obese as a result of prolonged exposure to HFD, do not exhibit changes in gonadotropin hormone levels. LH and FSH levels in OVX females were significantly higher than LH and FSH in unmodified females due to the lack of negative feedback (compare Figure 2B and Figure 2C), which confirms that ovariectomy was successful. However, neither LH nor FSH in OVX females were affected by diet (Figure 2C). Thus, there are profound sex differences and females are resistant to diet-induced changes in reproductive hormones. Additionally, these results demonstrate that ovarian hormones contribute to resistance to obesity, but are not necessary for the protection from hormonal changes exhibited by female mice.

**Figure 3:**
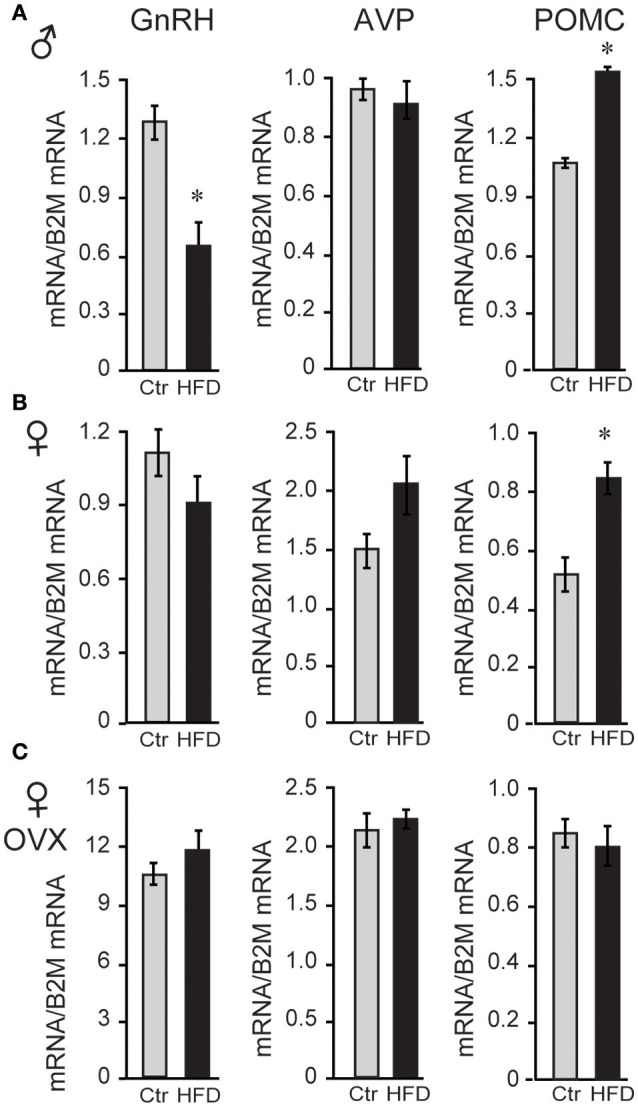
HFD elicits changes in hypothalamic neuropeptides expression. **(A)**, Male mice on HFD had 46% lower expression of *Gnrh* mRNA and 132% higher *Pomc* while *Avp* was not affected. **(B)**, Female mice did not exhibit changes in *Gnrh*, but had increased *Pomc* mRNA. **(C)**, There was no change in *Gnrh* expression in HFD compared to Ctr in ovariectomized (OVX) females, although *Gnrh* mRNA was higher in OVX females than unmodified females. *Indicates difference between Ctr (gray bars) and HFD (black bars), determined by Student's *T*-test followed by Tukey's HSD test.

Since LH levels are strictly regulated by GnRH neuropeptide from the hypothalamus, GnRH (*Gnrh*) expression was analyzed and revealed that male mice on HFD exhibited 46% reduced levels of *Gnrh* mRNA. To analyze specificity of repression, additional neuropeptides were assessed, and we determined that *Pomc* (POMC, proopiomelanocortin) mRNA was increased by 132%, while *Avp* (arginine vasopressin) mRNA was unchanged (Figure 3A). Neither unmodified or OVX female mice, on the other hand, exhibited diet-induced changes in *Gnrh* mRNA level, although OVX females exhibited ~10-fold higher *Gnrh* mRNA levels compared to unmodified females due to the lack of negative feedback (Figures 3B,C). These results again illustrate sex specific DIO-induced alterations in the neuropeptide gene expression in the hypothalamus, independent of the presence of ovarian hormones.

**Figure 4:**
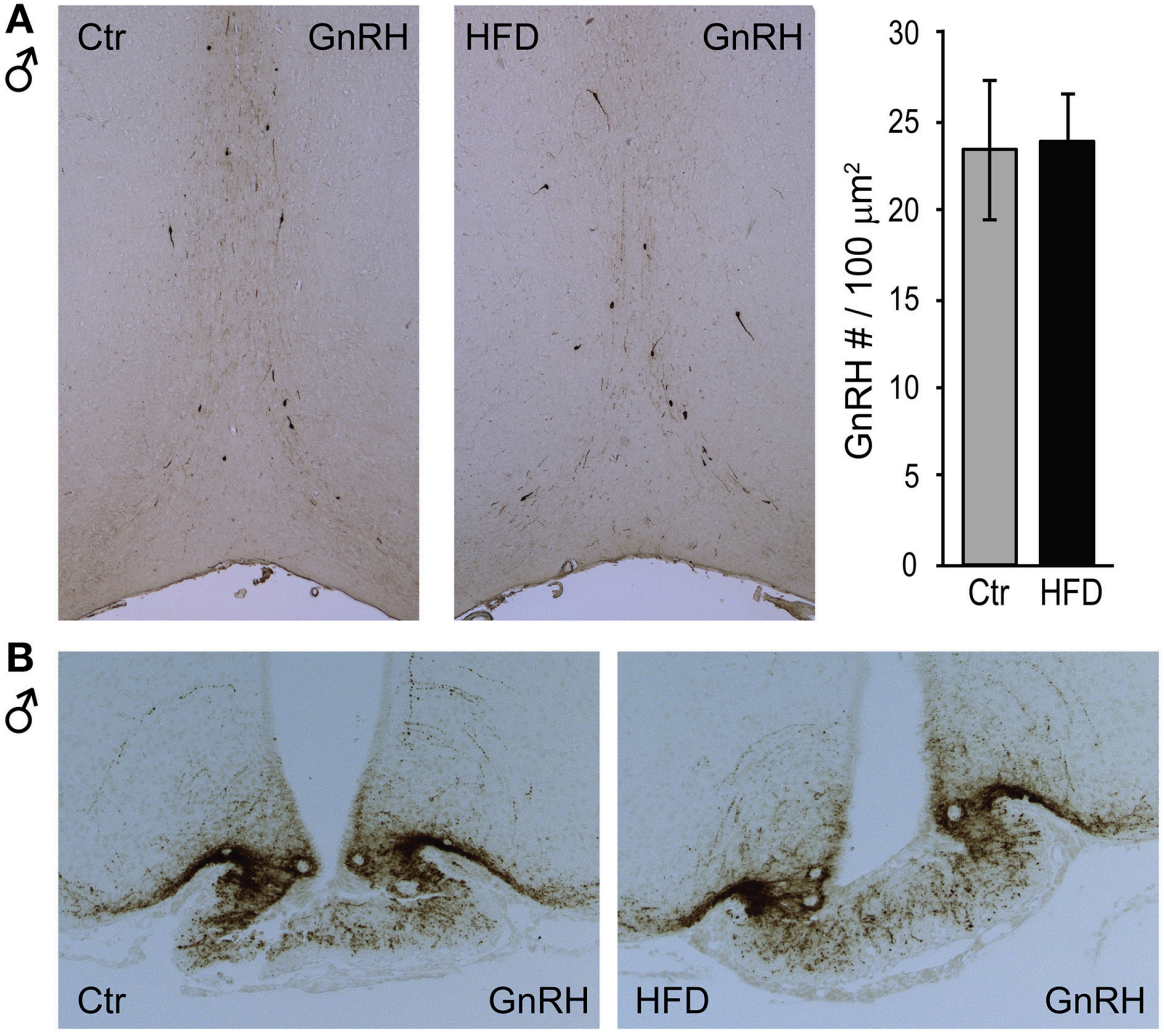
GnRH neuron number or axon targeting is unchanged in DIO mice. Coronal sections of the preoptic area (**A**, top) and median eminence (**B**, bottom) from Ctr and HFD fed male mice, were stained with anti-GnRH antibodies. No diet-mediated changes in GnRH neuron number, nor axon targeting were detected.

To determine whether a reduction in *Gnrh* mRNA levels in male mice stems from fewer GnRH neurons after exposure to HFD, we analyzed GnRH neuron number and detected no difference between diets (Figure 4A). We also detected no difference in response to diet in GnRH neuron axon targeting to the median eminence (Figure 4B). Thus, decrease in *Gnrh* mRNA levels correlates with the reduction in LH, testosterone and sperm count in male mice following HFD.

### Sex-specific differences in inflammatory cytokines in the circulation and the hypothalami of HFD-fed mice

**Figure 5:**
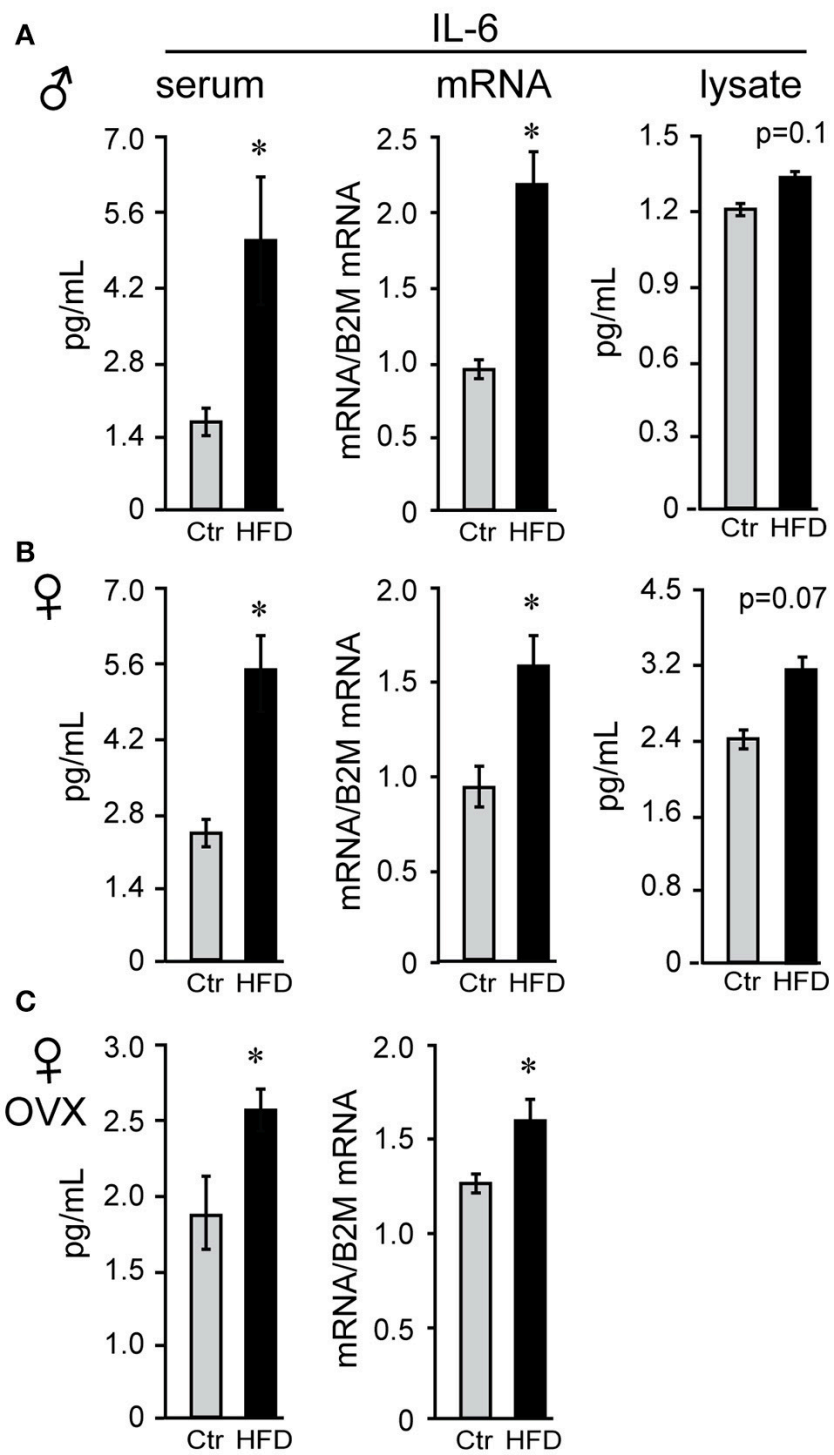
Interleukin-6 (IL-6) is increased in the serum and the hypothalamus of both male and female mice. Following Ctr (gray bars) and HFD (black bars) serum and hypothalami were collected from male **(A)**, unmodified female **(B)** and ovariectomized (OVX) female mice **(C)**. Cytokine levels in serum and hypothalamic protein lysates were measured using Luminex MagPix instrument and mouse ProcartaPlex 7 plex (Affymetrix eBioscience, San Diego, CA), while mRNA was assayed with qPCR. *Indicates significant difference (*p* < 0.05) between diets determined by Student's *T*-test and Tukey's posthoc comparison.

Obesity is characterized by hyperleptinemia, hyperinsulinemia, hyperlipidemia, hyperglycemia, and chronic inflammation. Significant effort has been devoted to determine the effects of increased leptin, insulin, fatty acids, or glycose on GnRH neuronal function without clear outcomes (13). We analyzed inflammatory cytokine levels following DIO in our mice and determined that inflammatory cytokines were elevated in the circulation as well as in the hypothalami of obese mice. There was no difference in serum levels of interleukin-6 (IL-6) between males, unmodified females or ovariectomized females on control diet (compare Figures 5A,B,C), and all three groups exhibited significantly elevated serum IL-6 following HFD. Increased IL-6 in the serum of HFD-fed male mice (Figure 5A) was previously demonstrated (57). IL-6 levels were also increased in the hypothalamic lysates, at the protein and mRNA levels, indicating that IL-6 was induced locally in the hypothalami of the DIO mice. Additionally, hypothalamic IL-6 was increased in both male and female mice (Figures 5B,C). However, increased cytokine levels were not detected in the cortex of neither male nor female mice (data not shown), indicating that neuroinflammation is specific for the hypothalamus.

**Figure 6:**
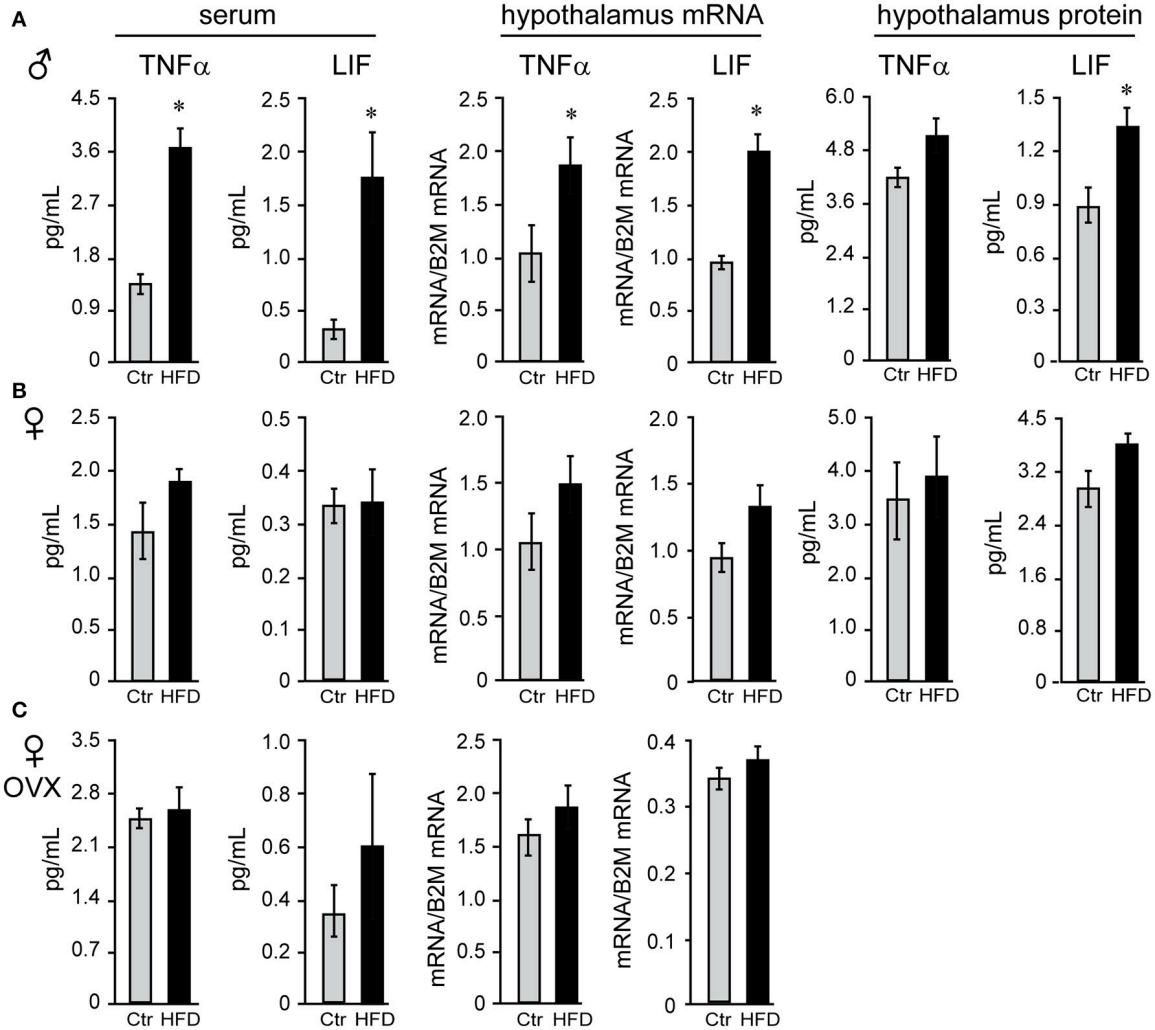
Tumor necrosis factor (TNF) α and leukemia inhibitory factor (LIF) are increased specifically in the male mice regardless of the presence of ovarian estrogens. Serum and hypothalami were collected from male **(A)**, unmodified female **(B)** and ovariectomized (OVX) female mice **(C)** following Ctr and HFD. Cytokine levels in serum and hypothalamic protein lysates were measured using Luminex MagPix instrument and mouse ProcartaPlex 7 plex (Affymetrix eBioscience, San Diego, CA), while mRNA was assayed with qPCR. Difference (*p < 0.05) between Ctr (gray bars) and HFD (black bars) were determined by Student's *T*-test followed by Tukey's HSD test.

We sought to identify cytokines that may exhibit sex differences in response to HFD. Tumor necrosis factor (TNF) α and leukemia inhibitor factor (LIF) were examined, since shown to increase in DIO (58). There was no sex difference in TNFα or LIF levels in serum of Ctr males and Ctr unmodified females, while removal of ovaries significantly increased TNFα concentration, but not LIF, in the serum of Ctr OVX females compared to unmodified Ctr females. TNFα and LIF increased specifically in male mice on HFD in the serum (3-fold or 300% and 4.83-fold or 483%, respectively), hypothalamic protein levels (112% and 155.6%, respectively) and mRNA expression levels in the hypothalamus (TNFα, 161% and LIF, 200%; Figure 6A), but were not changed in the cortex (data not shown); nor in unmodified or OVX female mice (Figures 6B,C). Therefore, IL-6 was increased in both sexes solely in the hypothalami, while TNFα and LIF are induced specifically in the hypothalami of male mice. Furthermore, while OVX females became susceptible to DIO, they remain protected from the increase in TNFα and LIF inflammatory cytokines in the hypothalamus.

**Figure 7:**
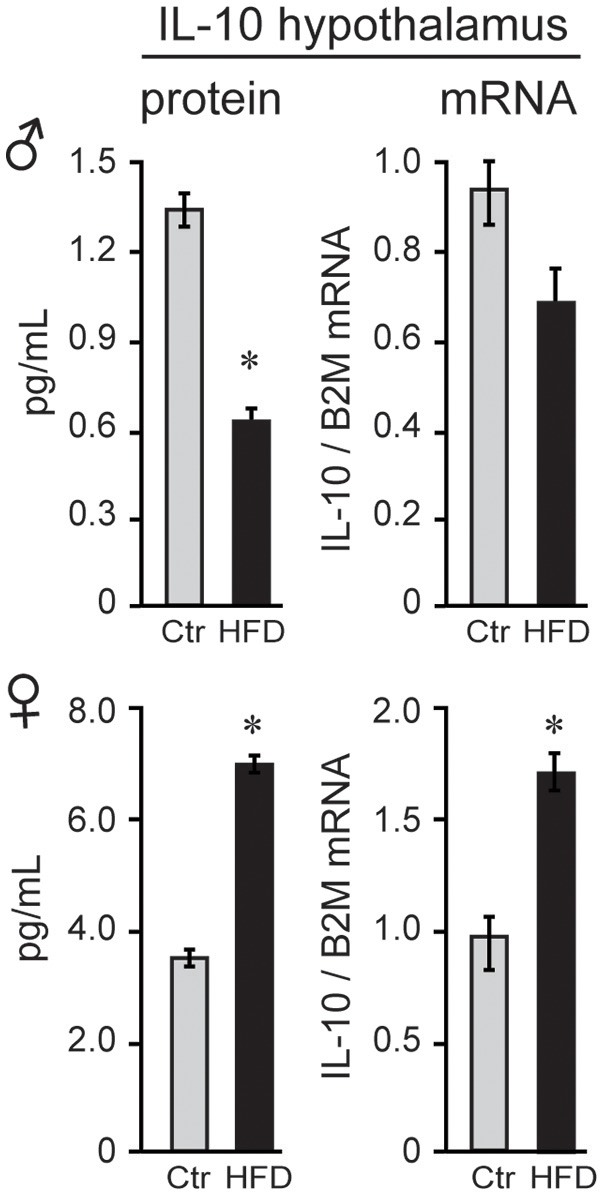
IL-10 is higher in female mice. IL-10 protein was higher in the hypothalami of female mice than male mice, and below detection in the serum in both sexes. After exposure to the HFD, IL-10, both protein and mRNA, decreased in males and increased in females. Statistical significance (*p < 0.05) between Ctr (gray bars) and HFD (black bars) were determined by Student's *T*-test followed by Tukey's test.

To explain possible protection of female mice, we analyzed anti-inflammatory cytokines. Female mice on Ctr diet exhibit significantly higher concentration of anti-inflammatory IL-10 than Ctr male mice (3.7 pg/ml female, 1.3 pg/ml male, Figure 7) in the hypothalamus, while IL-10 was below detection limit in the serum in both sexes. Exposure to HFD decreased level of IL-10 protein by 50.2% and *Il10* mRNA by 28% in male mice; however, HFD increased IL-10 protein to 188% and *Il10* mRNA to 165% in the female hypothalami (Figure 7). These results indicate that following HFD, inflammatory cytokines are increased, while anti-inflammatory cytokines are decreased in male hypothalami compared to the male mice on control diet. In contrast, female mice on HFD increase anti-inflammatory IL-10 concentration in their hypothalami, while lacking heightened inflammatory response.

### Regional differences in microglia activation in the hypothalamus

**Figure 8:**
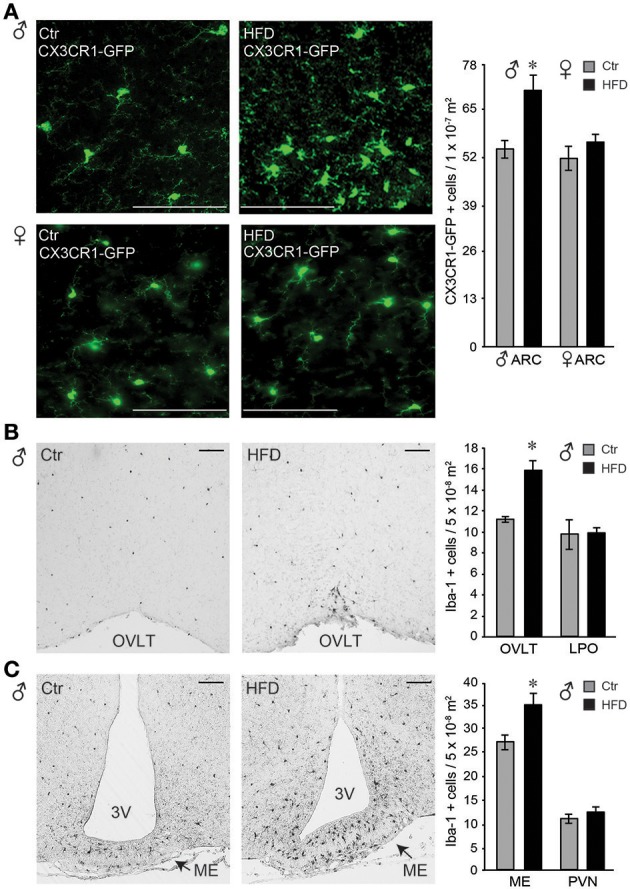
Iba-1 positive cells were more abundant in the circumventricular areas of the hypothalamus following HFD in male mice, but not in the female. **(A)**, Microglia, genetically labeled with CX3CR1-GFP, exhibit activated morphology in the arcuate nucleus after HFD specifically in male mice, but not in the female. Quantification determined that there is no difference in microglia numbers in Ctr males compared to Ctr females, or Ctr females compared to HFD females, while male mice on HFD had increased number of CX3CR1-GFP cells in the arcuate nucleus compared to the Ctr mice, indicated with an asterisk. **(B)**, Increased number of Iba-1 positive cells around the organum vasculosum laminae terminalis (OVLT) in DIO male mice fed HFD than Ctr mice, but the same numbers in the lateral preoptic area (LPO). **(C)**, Increased Iba-1 positive cell number in the median eminence (ME) and arcuate nucleus but not in the paraventricular nucleus (PVN) in male mice on HFD. **(A-C)**, Bar indicates 100 μm. Difference (*) between Ctr (gray bars) and HFD (black bars) were determined by ANOVA followed by Tukey's HSD test.

Microglia activation in response to the HFD in the arcuate nucleus of the mediobasal hypothalamus has been demonstrated previously (59–62). Using CX3CR1-GFP mice on Ctr and HFD, we also observed changes from ramified cell body with longer processes, to amoeboid, rounded cells body morphology with retracted processes, indicating activation of the microglia in the arcuate nucleus, (Figure 8A, top). These morphological changes were not observed in the female mice (Figure 8A, bottom). Quantification of CX3CR1-GFP positive cells demonstrated that there is no difference in the number of cells between Ctr males and Ctr females. Exposure to HFD increases the number of GFP-labeled cells in the arcuate nucleus in males, but not in females.

Identification of an increase in cell number in the arcuate nucleus in male mice on HFD prompted investigation of the regional changes in the number of Iba-1 positive cells (Iba-1, is expressed by the microglia in the brain and activated bone-derived macrophages). Increased number of Iba-1 positive cells was detected particularly around the organum vasculosum of the lamina terminalis (OVLT, Figure 8B) in the rostral hypothalamus and in the median eminence (ME) and arcuate nucleus in the mediobasal hypothalamus (Figure 8C), but not in the lateral preoptic area (LPO) or the paraventricular nucleus (PVN). Since PVN is located adjacent to the 3rd ventricle as is the arcuate nucleus, findings that PVN does not exhibit changes in Iba-1 positive cell number, while arcuate nucleus does, imply that the distance to the 3rd ventricle is not a contributing factor. Consistent with a lack of morphological changes, and lack of changes in the cell number in the arcuate nucleus, female mice did not exhibit any differences in Iba-1 cell number in either ME or OVLT hypothalamic area (data not shown). Therefore, solely in male mice, but not in female mice, HFD elicits an increase in Iba-1 positive cells in the circumventricular areas of the hypothalamus that are known to have a leaky blood-brain barrier.

### Infiltration of peripheral macrophages to the hypothalamus of obese male mice

**Figure 9:**
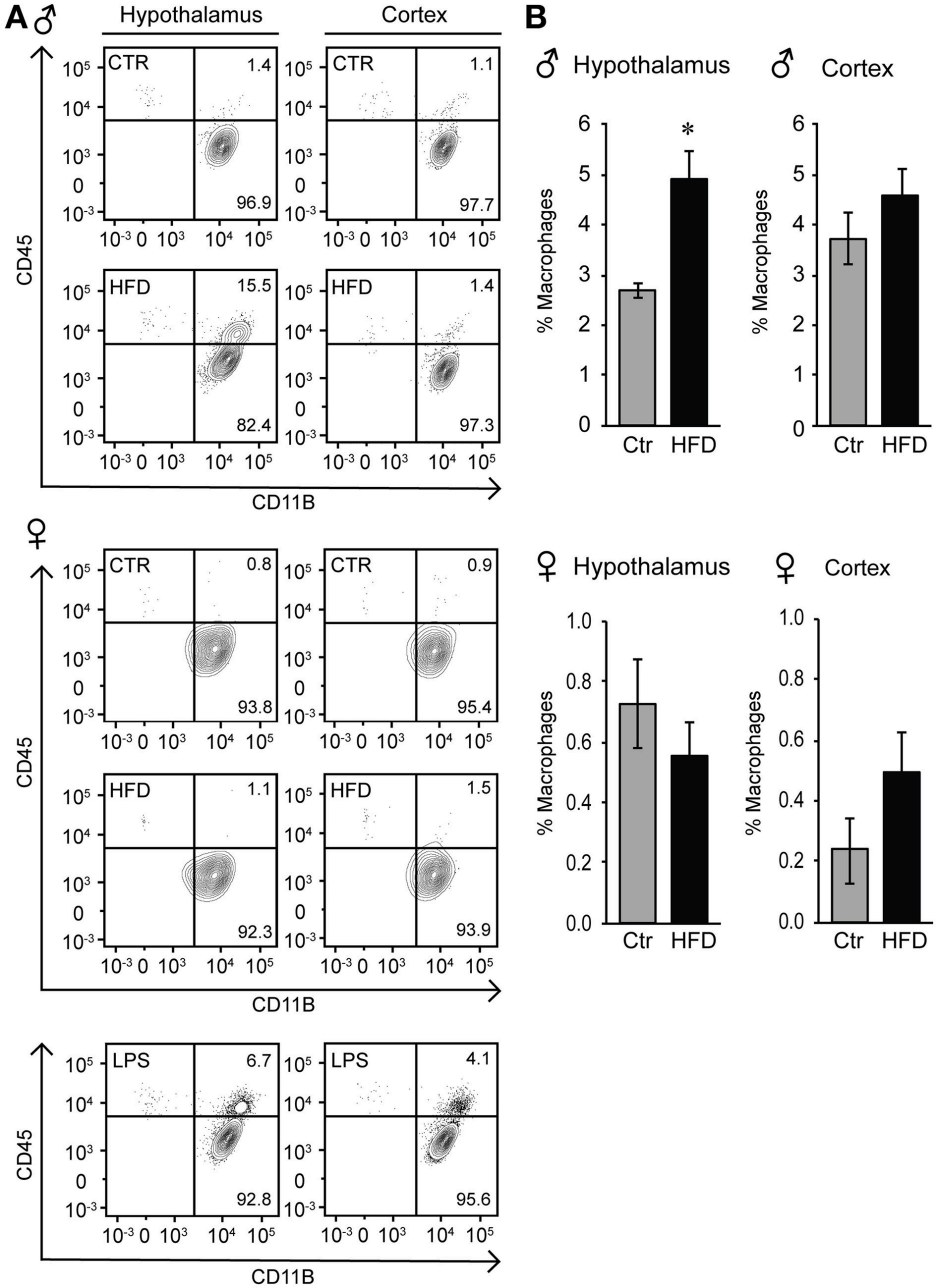
Infiltration of peripheral macrophages in the hypothalamus of the male mice on HFD but not female. **(A)**, Flow cytometry plot indicating presence of the CD45^high^ macrophage population that can be distinguished from the CD45^low^ microglia specifically in the hypothalami of HFD male mice, but not in the female. This population is not present in the cortex in either male or female mice. **(B)**, Quantification of A. Statistical significance (*) between Ctr (gray bars) and HFD (black bars) were determined by Student's *T*-test followed by Tukey's posthoc test.

Given that the increased Iba-1 cell numbers are observed in the areas that contain fenestrated capillaries, and that this increase is not dependent on the distance from the ventricle, we considered two possibilities, that either microglia sense the metabolic changes in the circulation that leads to their activation and proliferation, or that activated monocyte-derived macrophages enter the hypothalamus from the periphery, since increased adiposity leads to macrophage activation (33). Microglia and macrophages can be distinguished by flow cytometry from other CNS resident cells due to the presence of CD11b, and can be distinguished from each other by their differential expression of CD45, since microglia are CD11b+ CD45^low^ and macrophages are CD11b+ CD45^high^ (48, 63). Hypothalamus and prefrontal cortex were dissected, cells dissociated and subjected to flow analysis, which determined that macrophages are present in the hypothalami of obese male mice, but not in the cortex (Figure 9A). Consistent with the increase in inflammatory cytokines, macrophage recruitment to the hypothalamus is only noted in male mice, and is not observed in the female mice on HFD (Figures 9A,B).

**Figure 10:**
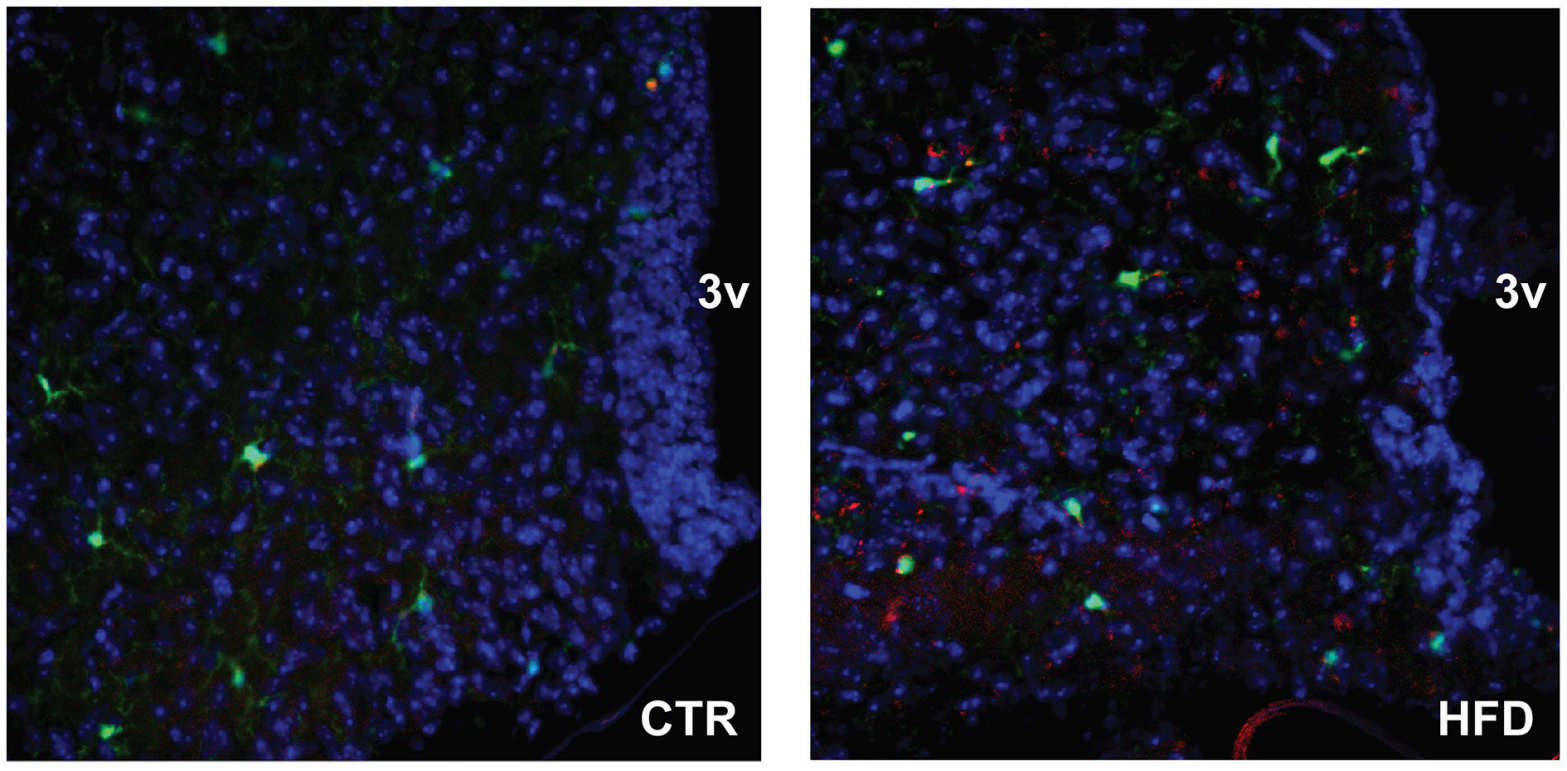
CCR2-positive macrophages localize to the parenchyma of the arcuate nucleus. Doubly fluorescent mice, where activated macrophages are genetically labeled red with CCR2-RFP and resident microglia are labeled green with CX3CR1-GFP, were placed on the control and HFD. Arcuate nuclei of mice on control diet contain only green fluorescence, while arcuate nuclei of mice on HFD contain green fluorescence of resident microglia, and red fluorescence indicating infiltration of peripheral macrophages.

To localize resident microglia and recruited monocyte-derived macrophages, we generated CX3CR1-GFP and CCR2-RFP double transgenic male mice. CX3CR1 is a fractalkine receptor expressed specifically by the microglia in the brain. CCR2 is a chemokine receptor involved in monocyte chemotaxis and infiltration; thus, the presence of RFP expression under CCR2-promoter control would indicate infiltrating activated macrophages. These mice were placed on a Ctr and HFD. In the Ctr, only green-labeled microglia positive for CX3CR1 are present in the arcuate nucleus of the mediobasal hypothalamus (Figure 10, coronal section with 3rd ventricle on the right side of the image), while in the HFD, RFP-labeled infiltrating macrophages are localized to the brain parenchyma in addition to the GFP-labeled resident microglia. Collectively, these results indicate that peripheral macrophage recruitment to the hypothalamic parenchyma following HFD only occurs in male mice.

### Decrease in GnRH neuron spines density in the hypothalamus of obese male mice

**Figure 11:**
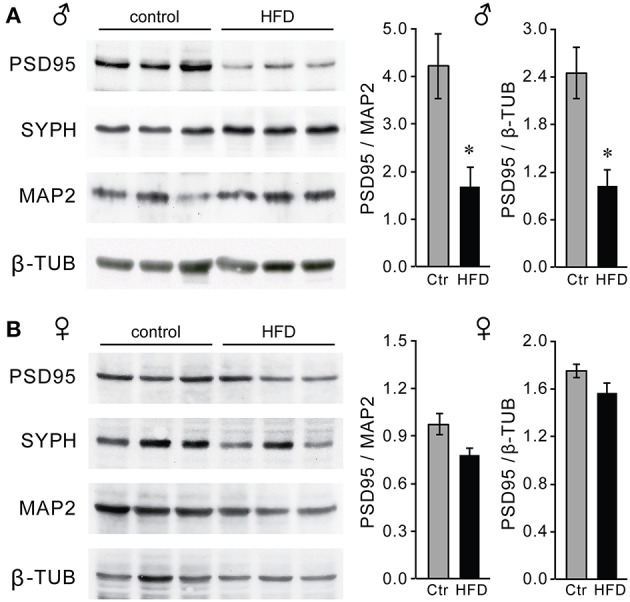
Decreased levels of PSD-95 synaptic protein in the hypothalami of male mice on the HFD. Western blot using hypothalamic lysates indicate lower levels of post-synaptic density protein 95 (PSD-95), but not of pre-synaptic protein synaptophysin (SYPH) in males **(A)** but not in females **(B)**. *Indicates statistical significance *p* < 0.05 determined by Student's *t*-test and Tukey's *post hoc* analysis, after quantification of PSD-95, neuronal marker MAP2 and housekeeping control β-tubulin.

Macrophage infiltration and elevated inflammatory cytokines in the hypothalamus may lead to synapse elimination (64–66); and subsequent dysregulation of GnRH neurons, which can explain reduced GnRH, LH and testosterone levels in male mice on the HFD. Given that we detected no changes in the number of GnRH neurons, we next determined if there are synaptic changes in the hypothalami of HFD male mice and specifically GnRH neurons. Western blot analyses revealed lower levels of excitatory post-synaptic density (PSD) 95 protein in the hypothalami of HFD male mice, while no changes were seen in the overall levels of the pre-synaptic protein synaptophysin (SYPH), usually detected in both excitatory and inhibitory pre-synaptic sites (Figure 11A). In contrast, PSD-95 and synaptophysin levels were unchanged in the hypothalami of female mice on HFD as compared to Ctr females (Figure 11B). Our results show changes in synaptic protein levels in the hypothalamus of male mice that are susceptible to DIO, but not neuro-inflammation resistant female mice.

**Figure 12:**
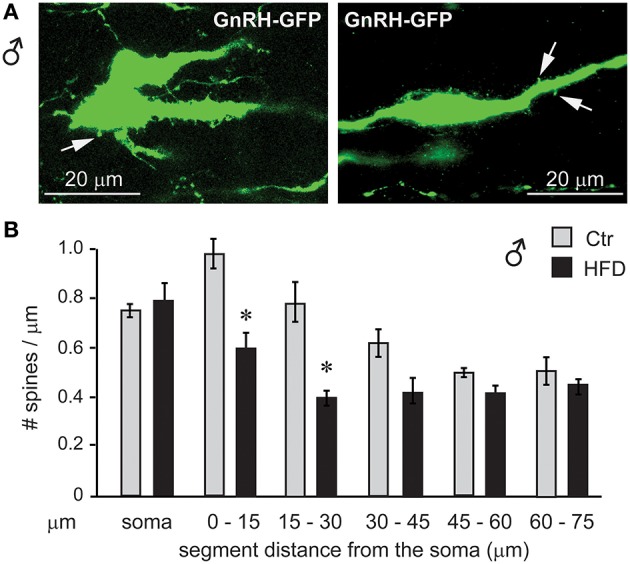
GnRH neuron spine density is lower in male mice on HFD. **(A)**, Coronal sections of the preoptic area in the hypothalamus of the GnRH-GFP mice following control and HFD were stained for GFP (green) to allow for spine count. **(B)**, Spines, identified as protrusions indicated with white arrows in **(A)**, were counted in the soma and in the visible length of the main axon in 15 μm intervals, which are indicated in the graph in comparison to the distance from the soma. *Indicates statistical significance *p* < 0.05 determined by one-way ANOVA followed by Tukey's *post hoc* analysis.

To assess synapses of specifically GnRH neurons, we placed GnRH-GFP mice (45) on the Ctr or HFD for 12 weeks and analyzed spine density, since changes in the number of spines have been linked to alterations in neuron connectivity (49–53). Spine density was analyzed in the GnRH-GFP neurons of the Ctr and HFD male mice, using the method described previously (54). Spines were identified as protrusions, 1–5 μm, from the soma or from the proximal axon of these mostly unipolar neurons (67). By scrolling through the z-stack obtained by confocal microscopy, we counted the spines on the soma and along the first 75 μm of the length of axon at 15-μm intervals. GnRH neurons from male mice on Ctr exhibited the same number of spines on the soma and in each of the 15 μm segments as determined before (55). GnRH neurons from HFD male mice exhibit fewer spines, especially in the region of the axon that is 45 μm proximal to soma (Figure 12). Therefore, obese male mice following HFD exhibit neuroinflammation and lower number of spines that may indicate reduced connectivity of the GnRH neurons, which in turn may lead to reduction in LH, testosterone and sperm numbers.

## Discussion

Herein we report several novel findings: (1) we identify sex differences underlying the neuroendocrine response to diet-induced obesity (DIO), and that ovarian hormones protect from DIO, but are dispensable for female resistance to neuropeptide and hormonal changes; (2) we show sex-specific differences associated with inflammatory cytokines, microglial activation and macrophages infiltration in the hypothalamus; in HFD male but not female mice; and (3) we posit a potential mechanism of obesity-induced impairment of hypothalamic function whereby obese males exhibit lower number of synaptic proteins, which may underlie negative effects of obesity on reproductive function.

Sex differences in response to DIO were reported before in mice and in rats (56). Based on these studies and observations in menopausal women it was hypothesized that a lack of estrogen increased adiposity, whereas estrogen replacement diminished it. Increase in adiposity following ovariectomy and removal of ovarian estrogen was observed in rodents (68, 69) and in monkeys (70). Our results concur that ovarian estrogen is protective from DIO, but do not support the assumption that ovarian estrogen is protective from hormonal and immunological changes. Hormonal and immune changes have not been examined in response to DIO in unmodified and ovariectomized (OVX) females before. It was assumed that hormonal changes would follow weight gain in females after ovariectomy, however our data indicate that is not the case. We demonstrate that females are protected from hormonal and immune changes regardless of the gonadal status.

Sex differences we observe may stem from variety of other factors, besides ovarian estrogen. In fact, extra-ovarian estrogen may play a role since the *Cyp19a1* gene, which encodes for aromatase that mediates estrogen synthesis, is expressed in other tissues, especially the brain and adipose. Brain-produced estrogen may regulate GnRH neuron function (71), while estrogen synthesis in adipose tissue may regulate deposition in various depots (72). There are also profound sex differences in the immune system (73), metabolic rate and oxidative phosphorylation (74), fat deposition (69) and adipocyte number and size (75), all of which may or may not be dependent on sex steroids, gonadally or locally produced. Our results indicate that sex differences may stem from differences in inflammation or alternate fat deposition, both of which may be interdependent. Females have higher prevalence of autoimmune diseases, but exhibit lower rates of infections and fewer chronic inflammatory diseases (73). Resistance to chronic inflammatory diseases may protect them from obesity-mediated chronic inflammation. Female mice in our study had elevated levels of anti-inflammatory IL-10 in the hypothalamus, which may provide them protection from neuroinflammation. On the other hand, we and others also determined differences in fat deposition (68, 76). Our results agree that males have larger visceral fat depots, or gonadal fat pad, with observation in other models and humans, and that premenopausal females preferentially deposit fat into the subcutaneous depot (56). Difference in fat deposition may also be sex steroid dependent (77, 78); and likely provides protection for females, since subcutaneous fat is less adversely correlated with negative effects of obesity than visceral fat. Variances in fat depots, not only absolute adiposity, may impact obesity induced inflammation, since visceral fat, more abundant in males, contains more infiltrating macrophages and higher expression of inflammatory cytokines (58, 79) This may increase male propensity for tissue inflammation, in other tissue including the brain.

A few studies have analyzed the effects of the high fat diet (HFD) on the gonadotropin hormones in mice concentrating on females, since they determined that females have longer estrous cycles, findings our results support (26, 80, 81). Due to the significant differences of various mouse strains to DIO, it is difficult to draw a direct comparison between some of these studies and ours, if other studies used a different strain or mixed strain mice (80). Several studies identified significantly different responses to HFD and some strains, such as BALBc and FVB, were surprisingly resistant to DIO (40). In DBA and C57BL/6J strains, that are prone to DIO, sex differences vary. Females of the DBA strain are prone to DIO, have lower pregnancy rates and lower *Gnrh* mRNA (39), while C57BL/6J females are resistant to DIO, as we have also shown herein. C57BL/6J mice, used in our study, are most often used in DIO studies since they develop insulin resistance and metabolic syndrome that matches human condition (42, 43). Reproductive hormones analyses in DIO C57BL/6J mice determined that diestrus females do not exhibit changes in LH, which agrees with our results (81). However, preovulatory levels of LH are lower following HFD, also aligning with the longer estrous cycles in our studies (26). While male mice exhibited lower FSH in both of our studies, employing the new ultra-sensitive assay we also detected decreased LH levels. Therefore, male mice have lower LH, testosterone and sperm count likely due to lower *Gnrh* mRNA and reduced number of spines in the GnRH neuron.

Lower *Gnrh* mRNA expression is a consistent finding in mice fed HFD. Repression of *Gnrh* mRNA in DIO mice has been reported previously [(26, 39), Supplemental Information]. Interestingly, another report analyzing obesity-induced genome-wide changes in the brain, detected GnRH as one of the most repressed genes (82). As stated above, acute cytokine infusion in the hypothalamus also represses *Gnrh* expression (36), suggesting that DIO repression of *Gnrh* mRNA may be mediated by increased cytokine concentration. Our studies agree that DIO diminishes *Gnrh* mRNA, and further demonstrate reduction in spine density in GnRH neurons. This may indicate that *Gnrh* expression may be regulated in an activity-dependent manner (83, 84), or alternatively, *Gnrh* gene may be repressed by cytokines independently of synaptic connectivity. Activity-dependent GnRH gene regulation by afferent neurons has been implied. Hypothalamic factors involved in reproductive function, such as RFamide-related peptide 3 (RFRP-3), a mammalian gonadotropin-inhibitory hormone ortholog; senktide, a neurokinin B receptor agonist; and oxytocin; elicit changes in LH serum levels, by altering both GnRH secretion and *Gnrh* transcription (85–87). On the other hand, GnRH neurons express cytokine receptors (88), and *Gnrh* may be repressed via activation of cytokine receptor signaling pathways.

Reduction in GnRH spine density may indicate lower neuron excitability. A decrease in synapses following HFD was reported previously in the arcuate nucleus in both NPY and POMC neurons, which are widely studied neuronal populations in response to DIO, since they comprise feeding and satiety circuitry in the hypothalamus (89). Specifically, elimination of inhibitory synapses on POMC neurons and excitatory synapses on NPY neurons was described. Reduction in inhibitory synapses may lead to increased POMC expression that was previously reported (89) and is consistent with our findings. Synaptic stripping, reduced levels of synaptic proteins and fewer spines following diet-induced obesity in male C57BL/6J mice were observed in hippocampal neurons as well (90). A decreased performance of HFD mice in cognitive tasks was attributed to the loss of synapses, fewer dendritic spines and a decrease in synaptic proteins in the prefrontal cortex (91). Since number of spines and the levels of PSD-95 synaptic protein are linked to neuronal connectivity, fewer spines and reduced levels of synaptic proteins, may indicate changes in neuronal activity. It was postulated that either estrogen induces changes in a number of synapses on POMC neurons, since sex differences were detected in the previous study as well (92); or that leptin is involved in synapse remodeling, since leptin is elevated in obesity and targets POMC neurons (89). Given that POMC and NPY neurons are located in the arcuate nucleus, in light of our results, their synapses may be also eliminated by macrophages or activated microglia. Microglia, brain resident immune cells, are involved in synapse prunning and synapse maturation during development (64) and in the regulation synaptic transmission and activity-dependent structural remodeling in adults (93). Peripheral, monocyte-derived macrophages, however, enter the brain in pathological conditions phagocytosing damaged cells and engulfing synapses (94, 95). Obesity-induced damage to POMC neurons that ultimately decreases POMC neuron number was postulated to elicit hypothalamic inflammation (96). We determined that GnRH neurons have fewer spines following HFD, without changes in GnRH neuron number. GnRH neuron cell bodies are located in the preoptic area around the OVLT, which has fenestrated capillaries, which allow for macrophage infiltration. POMC and NPY neurons are located in the arcuate nucleus, which is dorsal to the median eminence that also contains fenestrated capillaries. Thus, specificity in synaptic elimination may stem from the location of the neurons and proximity to the circumventricular areas with leaky blood brain barrier that allows for macrophage infiltration.

Previous studies analyzing effects of obesity identified that neuroinflammation is specific for the hypothalamus and determined that microglia changes morphology specifically in the arcuate nucleus (59, 62). In agreement, our studies also failed to detect microglia morphology changes in the cortex and detected them specifically in the hypothalami. Sex differences in microglia activation in response to obesity have also been reported previously (97). Our studies similarly detected morphology changes specifically in the male mice. To assess the regional specificity, we further analyzed other areas besides arcuate nucleus and determined that microglia morphology changes around other circumventricular areas with fenestrated capillaries, such as OVLT. We considered two possibilities, that either microglia sense metabolic changes in the circulation via fenestrated capillaries in circumventricular areas or that increased numbers indicate that peripheral cells which label with anti-Iba-1 cross into the brain parenchyma specifically in these areas due to the lack of the blood-brain barrier. That prompted us to investigate potential peripheral cells and determine that monocyte-derived macrophages enter the hypothalamus.

Macrophages are functionally dominant cells in obesity-induced chronic inflammation (98). With increased adiposity, proportion of macrophages in adipose tissue increases from 10 to 50%, and they change from less inflammatory M2 phenotype to pro-inflammatory M1 phenotype (33, 58, 99, 100). Elevated macrophage infiltration and cytokines secretion is more highly correlated with visceral adiposity (56). Inflammatory cytokines secreted by the adipose tissue macrophages contribute to the increase in TNFα and IL-6 in the circulation and chronic inflammation in obese mice and people (34). We determined that activated macrophages infiltrate the hypothalamus in the DIO male mice. Macrophages are recruited to adipose tissue in part via CCR2 interaction with CCL2 that is upregulated in obesity (101). CCR2 is involved in the macrophage recruitment to the liver in obese mice, which contributes to insulin resistance (102). Thus, macrophages activated by increased adiposity infiltrate parenchyma of other tissues. We demonstrated that macrophages recruited to the hypothalamus of obese mice likewise express CCR2. Macrophage switch to pro-inflammatory phenotype with increasing obesity also entails a decrease in the anti-inflammatory IL-10 expression in obese adipose tissue (32, 103). Thus, protective role of IL-10 in adipose tissue in DIO has been postulated previously but sex differences were not examined (104, 105). We determined that female mice have higher basal levels of IL-10 in the hypothalamus and that obesity exacerbates sex differences since IL-10 is further decreased in obese males in the hypothalamus as well. In the brain, IL-10 is expressed by glial cells (106) and has a protective function (107). We postulate that females, due to the need to handle larger weight changes during pregnancy and lactation, exhibit higher levels of protective anti-inflammatory IL-10 and that increased levels of anti-inflammatory cytokines in female may afford them protection from obesity-induced inflammation.

We demonstrated that male mice, but not females, on HFD exhibit macrophage infiltration to the hypothalamus in addition to the microglia activation, however future studies will identify which of these cells is activated first. Our studies also demonstrate that synaptic proteins are decreased in the hypothalami of obese male mice, and that specifically GnRH neurons have fewer spines and lower *Gnrh* mRNA expression. Diminished *Gnrh* mRNA transcription and reduction in GnRH neuron activity may contribute to lower LH levels, reduced testosterone and diminished sperm numbers. Previous studies analyzing crosstalk between metabolism and reproductive function determined that GnRH neurons may integrate obesity-induced changes in glucose directly (108), but changes in insulin (29) and leptin (28, 30) are most likely relayed indirectly via neuronal afferents that synapse to GnRH neurons. In this manuscript, we show that GnRH neurons may also mediate the effects of obesity-induced synaptic changes on reproductive function. Future studies will determine if synaptic changes result in changes in the secretion of the neuropeptide that regulates gonadotropin hormone levels and reproduction. Furthermore, we report sex specific changes in neuroinflammation and fat deposition that may explain sex differences in adverse effects of obesity. Future studies will further elucidate sex differences in adipose tissue accumulation and obesity-induced inflammation. Herein, we combine hormonal and immune parameters to identify sex-specific effects of obesity on neuroendocrine function.

## Author contributions

NL: performed most of the experiments; CJ: performed some experiments; MN: provided expertise in flow analyses and edited the manuscript; IE: provided expertise in neuron and dendritic spine count; EW: provided expertise in flow cytometry and edited the manuscript; MJC: provided expertise in microglia biology, supplied transgenic mice used in the study and revised the manuscript; DC: conceived the experiments and wrote the manuscript.

### Conflict of interest statement

The authors declare that the research was conducted in the absence of any commercial or financial relationships that could be construed as a potential conflict of interest.
